# Socioeconomic Impact, Equity, and Sustainability in Head and Neck Cancer Surgery: A Structured Narrative Review

**DOI:** 10.3390/epidemiologia7040088

**Published:** 2026-06-23

**Authors:** Francesco Chiari, Salvatore Ferlito, Guglielmo Piccione, Rodolfo Modica, Mario Lentini, Giancarlo Carmelo Botto, Salvatore Maira, Skander Kedous, Carlos Chiesa-Estomba, Pierre Guarino, Jerome Rene Lechien, Antonino Maniaci

**Affiliations:** 1 Otolaryngology Head and Neck Unit, “Santo Spirito” Hospital, 65121 Pescara, Italy; francesco.chiari.med@gmail.com (F.C.); pierreguarino@hotmail.com (P.G.); 2 Department of Medical, Surgical Sciences and Advanced Technologies G.F. Ingrassia, University of Catania, 95123 Catania, Italy; ferlito@unict.it; 3 Ospedale Maggiore Modica, ASP 7 di Ragusa, 97100 Ragusa, Italy; guglielmo.piccione@asp.rg.it (G.P.); rodolfo.modica@asp.rg.it (R.M.); mario.lentini@unikore.it (M.L.); giancarlocarmelo.botto@asp.rg.it (G.C.B.); 4 Department of Medicine and Surgery, “Kore” University of Enna, 94100 Enna, Italy; salvatore.maira@unikore.it; 5 Ears Nose and Throat, Head and Neck Surgery Department, Salah Azaiz Institute, Tunis 1000, Tunisia; skander.kedous@fmt.utm.tn; 6 Department of Otolaryngology-Head and Neck Surgery, Donostia University Hospital, 20014 San Sebastián, Spain; chiesaestomba86@gmail.com; 7 Faculty of Medicine, Deusto University, 48007 Bilbao, Spain; 8 Head and Neck Research Committee, Young-Otolaryngologists of the International Federations of Oto-Rhino-Laryngological Societies (YO-IFOS), 70123 Paris, France; jerome.lechien@umons.ac.be; 9 Department of Innovative Technologies in Medicine & Dentistry, University “G. d’Annunzio”, Chieti-Pescara, 66100 Chieti, Italy; 10 Department of Surgery, UMONS Research Institute for Health Sciences and Technology, University of Mons (UMons), 36100 Mons, Belgium

**Keywords:** head and neck cancer, sustainability, workforce, telemedicine, artificial intelligence, 3D printing, health policy, global surgery

## Abstract

Background: Sustainable head and neck cancer (HNC) surgery is challenged by environmental impact, workforce shortages, inequitable access to advanced techniques, and policy constraints. Addressing these areas is critical for equitable, high-quality care. Methods: This structured narrative review synthesizes evidence on environmental sustainability, workforce development, technological innovation, health policy, and socioeconomic determinants in HNC surgery, without aiming to provide a systematic or exhaustive evidence synthesis. Sources included peer-reviewed literature, global workforce surveys, and international policy reports, with a focus on disparities between high-income countries (HICs) and low- and middle-income countries (LMICs). Results: Operating rooms produce up to 70% of hospital solid waste and consume 3–6 times more energy than other units; reusable instruments and improved waste segregation can reduce carbon footprints by over 50%. Workforce shortages are severe in LMICs, where subspecialty training is scarce; global partnerships, bidirectional education, and simulation-based learning can expand local capacity. Telemedicine, artificial intelligence, and three-dimensional printing enhance surgical planning, training, and access but may widen disparities without equitable deployment. Policy tools—including diagnosis-related groups, bundled payments, and universal coverage—affect access and innovation uptake. Pandemic preparedness underscores the value of resilient systems with flexible staffing and telehealth integration. Conclusions: HNC surgery requires coordinated action across environmental, workforce, technological, socioeconomic, and policy domains; however, future systematic reviews are needed to comprehensively map the evidence base and assess its methodological quality. Embedding sustainability in clinical practice, ensuring equitable innovation access, and aligning reimbursement with high-value care can strengthen system resilience, improve outcomes, and support long-term surgical service viability.

## 1. Introduction

Head and neck cancer (HNC) surgery has an essential position across global health systems owing to the nexus of oncologic outcomes, economic sustainability, environmental stewardship and health-system organization. As a major global health burden, there are more than 660,000 new cases of HNC each year, and it results in nearly 325,000 deaths worldwide [1,2]. Beyond mortality and morbidity, the multidisciplinary nature of managing these conditions—encompassing surgery, radiotherapy, chemotherapy, reconstructive procedures, and intensive rehabilitative services—places considerable demands on resources, driving up both direct and indirect costs of care [3,4].

Although interdependent across the entire HNC treatment spectrum, we focused our review on surgery as operative care is still one of the most resource-intensive and health system-dependent components of HNC management, spanning environmental sustainability, workforce capacity, infrastructure needs, and peri-operative policy design. Sustainability in HNC surgery is defined as the capacity of surgical systems to deliver timely, equitable, high-quality, and financially viable cancer care while minimizing avoidable environmental impact and preserving workforce, technological, and institutional resilience over time. Accordingly, it is not limited to environmental stewardship, nor is it treated as a set of parallel independent domains. Rather, it is conceptualized as an interacting health-system construct in which workforce capacity, geographic access, reimbursement structures, responsible technological adoption, resource consumption, and patient-level outcomes mutually influence one another. Limited workforce density may increase travel burden and treatment delay. Reimbursement structures may determine whether advanced technologies are equitably adopted. In addition, environmental strategies may be feasible only when procurement, sterilization, and institutional infrastructure are adequately supported.

Mounting evidence from systematic reviews underscores the immense economic burden posed by HNC. In high-income countries such as the United States (US), annual direct medical expenditures for HNC reached approximately US $3.6 billion in 2010, with indirect costs—primarily due to lost productivity and premature mortality—approaching similar levels [5,6]. Per-patient costs are likewise substantial; estimates from commercially insured cohorts suggest five-year cumulative healthcare expenditures exceeding US $79,000, compared to US $45,000 among matched controls without HNC [3,4]. In Europe, data also demonstrate elevated costs associated with surgical and systemic interventions, although methodological variability and the scarcity of prospective studies complicate cross-country comparisons [7,8].

The financial strain often persists into survivorship: patients frequently experience “financial toxicity” as the long-term costs of follow-up, functional rehabilitation, secondary effects, and lost income accumulate [9]. Such burdens undermine quality of life and adherence to ongoing care, further exacerbating socioeconomic disparities. Individuals from lower-income or minority populations are disproportionately affected, facing delays in diagnosis, reduced access to advanced surgical care, and poorer functional and oncologic outcomes—a reflection of broader health inequities intrinsic to HNC surgery [10,11]. Concurrently, healthcare systems face an urgent imperative to balance clinical excellence with environmental responsibility. Operating theaters, particularly those used for HNC surgery, are high-impact areas for both energy consumption and waste generation. Operating rooms consume three to six times more energy than the hospital average and produce up to 70% of hospital waste [12]. In HNC contexts, the carbon footprint of single-use items—such as surgical drapes and gowns—can be considerable. For example, disposable drapes for tonsillectomy procedures alone may generate approximately 840 g of CO_2_ equivalents per case, prompting initiatives to adopt reusable alternatives that reduce both emissions and costs [13]. The rationale for adopting sustainable practices thus aligns ecological goals with economic imperatives. Interventions such as replacing disposable surgical gowns with reusable ones, optimizing instrument trays, recycling materials, and phasing out carbon-intensive anesthetic gases like desflurane offer opportunities to meaningfully reduce emissions while generating cost savings [14]. Early adopters in Scotland and North America have eliminated desflurane altogether, achieving both environmental and financial benefits. However, broader systemic changes—including procurement reforms, supply-chain adjustments, and improved energy management—are still required within HNC surgical practice [15,16,17,18]. Adding to the complexity, HNC surgical care requires high-level technical expertise, extensive multidisciplinary collaboration, specialized infrastructure, and long-term follow-up to monitor oncologic and functional outcomes [1]. In resource-limited settings—particularly across low- and middle-income countries (LMICs)—shortages of trained personnel, surgical materials, and rehabilitative services amplify both socioeconomic inequities and environmental vulnerabilities [2]. Ensuring the sustainability of workforce capacity, equipment supply, and institutional resilience is therefore integral to improving care quality and ensuring equitable access.

Accordingly, this review does not attempt to generate pooled estimates or a unified cost–utility model but instead organizes heterogeneous evidence into clinically relevant domains. By synthesizing evidence on epidemiologic burden, economic impact, disparities in access, cost-effectiveness of interventions, and environmentally responsible practices, we aim to propose integrative models that align clinical excellence with fiscal prudence and environmental stewardship. This study is a structured narrative review, which synthesizes core determinants of the sustainability of head and neck cancer surgery and analyzes these factors as interactive components within a generalized health system framework.

## 2. Materials and Methods

### 2.1. Search Strategy and Literature Identification

This structured narrative review was conducted in accordance with methodological guidance for narrative reviews and narrative synthesis in healthcare research, including recommendations by Green et al. for narrative literature reviews and the SANRA framework for assessing the quality of narrative review articles [19,20].

The search strategy was broad and structured but not exhaustive in the manner of a systematic review; therefore, no PRISMA flow diagram, formal protocol registration, or quantitative evidence pooling was planned.

A comprehensive literature search was performed across multiple electronic databases including PubMed/MEDLINE, Scopus, Web of Science, Cochrane Library, and Google Scholar from inception through January 2025. The search strategy employed a combination of Medical Subject Headings (MeSH) terms and free-text keywords encompassing three primary domains:

(1) HNC surgery economics (“head and neck neoplasms”, “otolaryngology”, “cost-benefit analysis”, “healthcare costs”, “economic burden”);
(2) healthcare sustainability (“sustainable development”, “carbon footprint”, “medical waste”, “green surgery”);
(3) healthcare disparities (“healthcare disparities”, “socioeconomic factors”, “health equity”, “access to healthcare”).

Boolean operators were utilized to combine search terms systematically, with the primary search string structured as: (“head and neck surgery” OR “otolaryngology” OR “head and neck cancer”) AND (“economic*” OR “cost*” OR “financial” OR “sustainability” OR “environmental” OR “carbon” OR “waste”) AND (“disparit*” OR “equity” OR “access”).

The search strategy was broad and structured but not exhaustive in the manner of a systematic review; therefore, no PRISMA flow diagram, formal protocol registration, or quantitative evidence pooling was planned. Additional targeted searches were conducted for specific subtopics including surgical waste management, energy consumption in operating theaters, and cost-effectiveness analyses of HNC interventions.

### 2.2. Inclusion and Exclusion Criteria

Studies were included if they met the following criteria: addressed economic aspects, environmental impact, or socioeconomic disparities in head and neck surgery; were published in peer-reviewed journals; available in English, Spanish, French, German, or Portuguese; included primary data, systematic reviews, meta-analyses, or comprehensive narrative reviews. Gray literature sources, including governmental reports, professional society guidelines, and policy documents from organizations such as the World Health Organization (WHO), were also incorporated when providing unique insights into global health perspectives. Exclusion criteria encompassed: case reports or small case series with fewer than 10 patients; conference abstracts without full-text availability; opinion pieces or editorials without systematic evidence synthesis; studies focusing exclusively on pediatric populations unless addressing broader sustainability or economic themes; publications predating 1990 for economic analyses due to substantial healthcare system changes, though seminal historical works were retained for contextual understanding.

The final reference list comprised 56 sources selected for relevance to the predefined thematic domains. Because this review was designed as a structured narrative review rather than a systematic review, records were not prospectively screened or tracked using a PRISMA-style flow process, and the findings should not be interpreted as an exhaustive mapping of all socioeconomic determinants in HNC surgery. It built an explanatory analytical framework to clarify the interaction logic of influencing factors throughout the full cycle of head and neck cancer surgery.

### 2.3. Data Extraction and Synthesis

A standardized data extraction framework was developed to capture key information including study design, geographic setting, population characteristics, economic outcomes, environmental metrics, and equity considerations. For economic studies, data on direct medical costs, indirect costs, cost-effectiveness ratios, and financial toxicity measures were systematically recorded. Environmental sustainability studies were evaluated for carbon footprint assessments, waste generation metrics, energy consumption data, and intervention outcomes. A structured narrative review was selected because the evidence base included highly heterogeneous study designs, populations, settings, and outcome measures. The included literature addressed direct medical costs, indirect societal costs, financial toxicity, environmental metrics, workforce capacity, digital innovation, and policy determinants. These domains are not sufficiently homogeneous to support meta-analysis or a single pooled quantitative estimate. A narrative synthesis was therefore considered more appropriate for integrating diverse evidence and developing a conceptual framework for sustainability in HNC surgery. Instead, the included literature was appraised narratively according to study design, transparency of methods, population or setting, relevance to HNC surgery, and consistency with the thematic domains of the review. Instead, the included literature was appraised narratively according to study design, transparency of methods, population or setting, relevance to HNC surgery, and consistency with the thematic domains of the review. The high-level and medium-level custom classification descriptors set forth in this paper only assess study relevance and robustness and are in no way risk of bias ratings, GRADE ratings, or certainty of evidence ratings.

Studies were grouped thematically according to primary focus areas: epidemiologic and economic burden, disparities in access and outcomes, cost-effectiveness of interventions, environmental sustainability initiatives, and integrated sustainability models.

### 2.4. Analytic Framework

The synthesis was guided by a systems-level conceptual framework recognizing that sustainability in HNC surgery is shaped by interacting determinants across patient, institutional, health-system, and societal levels. This framework was selected because the included literature spans heterogeneous domains, including economic burden, financial toxicity, geographic access, workforce capacity, environmental impact, digital innovation, reimbursement, and health-system resilience. These domains cannot be reduced to a single endpoint or pooled summary metric, but they can be organized according to their role within surgical care delivery. At the structural level, socioeconomic status, geography, insurance coverage, referral pathways, and national health policy influence access to specialist HNC services. At the institutional level, workforce distribution, surgical volume, infrastructure, procurement, sterilization capacity, digital readiness, and environmental practices determine the feasibility and quality of care delivery. At the innovation and policy level, reimbursement structures, telehealth, artificial intelligence, three-dimensional printing, and value-based care models may improve efficiency and access but can also widen disparities when implemented without equity safeguards. These determinants ultimately influence patient-level outcomes, including time to diagnosis, time to treatment, access to advanced surgery, perioperative morbidity, survivorship burden, financial toxicity, quality of life, and long-term continuity of care.

The review was organized around five thematic domains: (1) epidemiologic and economic burden, (2) resource allocation and cost-effectiveness, (3) environmental and institutional sustainability, (4) workforce and capacity building, and (5) innovation, reimbursement, and system resilience. The purpose of this framework was not to generate a formal causal model or a unified cost–utility estimate but to support conceptual integration across heterogeneous evidence and to identify health-system mechanisms that may be tested in future systematic reviews, registry-based studies, and implementation research.

## 3. Results

The results are presented as a thematic synthesis rather than as a systematic enumeration of all available studies. Each section highlights representative evidence and uses it to define key domains relevant to sustainability in HNC surgery.

### 3.1. HNC Surgery Economics

**Figure 1:**
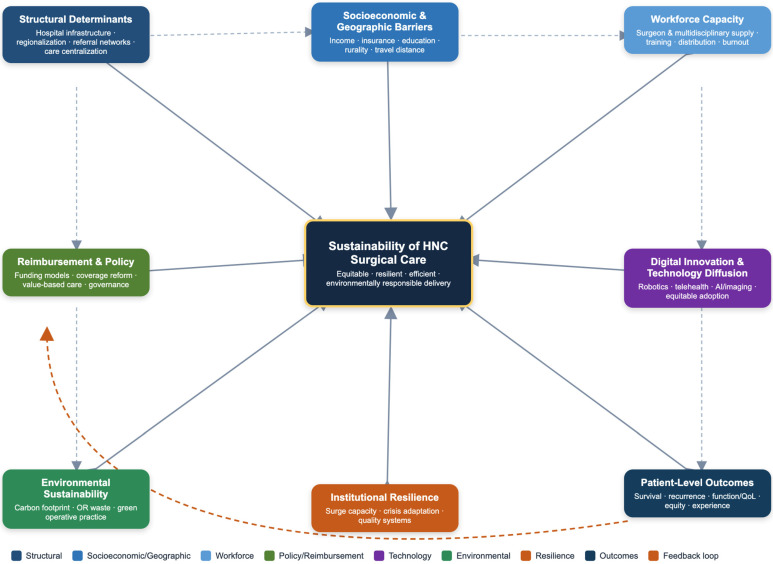
Conceptual model of sustainability in head and neck cancer (HNC) surgery. Rather than depicting a unidirectional cascade of disparities, the model situates sustainability of surgical care at the center of a dynamic system in which structural determinants, socioeconomic and geographic barriers, workforce capacity, reimbursement and policy, digital innovation and technology diffusion, environmental sustainability, and institutional resilience interact bidirectionally and exert cross-domain influence on one another (dashed arrows). These interactions converge on patient-level outcomes (survival, recurrence, function/quality of life, equity, and experience), which in turn inform policy and structural adaptation through a feedback loop, underscoring the iterative, self-reinforcing nature of a sustainable surgical care system.

A multilevel framework of the main disparities affecting HNC surgery is outlined in Figure 1, which presents the system-level conceptual model for head and neck cancer surgery. Structural, socioeconomic, workforce, technological, environmental, reimbursement, and policy determinants interact to influence access, outcomes, and long-term sustainability in HNC surgery.

Disparities in HNC surgery arise across interconnected levels, including epidemiologic burden, delayed diagnosis, geographic barriers to specialist referral, workforce limitations, limited availability and access to advanced technologies as well unequal reimbursement and policy support. These pathways are not independent of each other but interact to determine stage at presentation, time to treatment, postoperative course, survivorship, and long-term socioeconomic impact. The diagram additionally highlights critical points of intervention, including strengthening referral networks, increasing the training of the workforce, implementing context-appropriate digital health tools, improving financial protection, and integrating environmentally sustainable–surgical practices. That is why consider all of these aspects in one conceptual model clarifies that sustainability in HNC surgery can be achieved only by a multi-dimensional and system-level approach. In this review, the term “disparities” refers to inequalities in access to specialist care, timeliness of diagnosis and treatment, workforce availability, access to advanced technologies, financial protection, and institutional capacity to deliver sustainable surgical care.

#### 3.1.1. Epidemiology and Economic Burden of Head and Neck Diseases

The included literature consistently identifies HNC as a major source of clinical and economic burden worldwide, with marked variation across regions, disease subtypes, and access-to-care settings. Each year, more than 890,000 new cases of head and neck cancer are diagnosed worldwide, leading to approximately 450,000 deaths and placing these malignancies among the ten most common cancers globally [21]. Oral cavity cancers alone accounted for over 355,000 new cases and 177,000 deaths in 2018, with the highest incidence observed in South Asia and parts of Latin America [5]. Epidemiologic patterns vary markedly: nasopharyngeal carcinoma remains endemic in East and Southeast Asia due to genetic susceptibility and the high prevalence of Epstein–Barr virus, whereas human papillomavirus-positive oropharyngeal squamous cell carcinoma has risen sharply in Western populations [22]. Beyond malignancy, traumatic injuries to the head and neck—particularly those resulting from road traffic accidents, occupational hazards, and interpersonal violence—are major contributors to the global surgical burden. Traumatic brain and cervical spine injuries account for a disproportionate share of long-term disability and healthcare utilization, especially in low-resource settings where trauma systems remain underdeveloped [2].

The direct costs of head and neck diseases are considerable. In the US, per-patient direct medical expenditures for head and neck cancer management can exceed US $79,000 over five years, covering surgery, chemoradiotherapy, imaging, hospitalization, and long-term follow-up [3,4]. In Europe, Sweden has estimated its national direct healthcare cost for HNC at approximately €92 million annually [7].

Indirect costs—including lost productivity, caregiver time, and premature mortality—often surpass direct medical costs. In Sweden, economic models suggest that up to 64% of the total financial burden of head and neck cancer stems from lost productivity, amounting to more than €59 million annually [7]. Similarly, road traffic trauma—frequently involving maxillofacial and cervical injuries—generates indirect costs estimated between C$4.3 billion and C$62.9 billion annually in Canada [23]. In the US, the cost of managing laryngotracheal stenosis is comparable to that of chronic conditions such as diabetes, underscoring the persistent systemic strain posed by non-malignant head and neck conditions [4].

The global economic impact of HNC is profound. Between 2018 and 2030, these malignancies are projected to account for more than US $535 billion in lost economic output worldwide, primarily due to workforce attrition and premature deaths [1]. LMICs are disproportionately affected: Southeast Asia and Oceania are projected to lose over US $180 billion and South Asia an additional US $133 billion during this period [1].

From the patient’s perspective, financial toxicity is a pervasive issue. In LMICs, cancer patients often spend over 40% of their annual household income on out-of-pocket medical expenses, including transportation, co-payments, and supportive care [24]. Even in high-income countries (HIC), indirect financial pressures are associated with delayed treatment initiation, reduced adherence, increased distress, and lower quality of life [4,8,9].

In both HICs and LMICs, these indirect effects often reach beyond the health system, influencing employment continuity, household income, caregiver labor force participation and long-term social functioning. Consequently, lost productivity and financial toxicity should be considered key components of the ‘real-world’ burden of HNC on patients alongside conventional estimates of direct hospital spend and reimbursement. These inequities in financial burden are closely intertwined with disparities in access to care and health outcomes. Regions with low surgical workforce density report higher case-fatality rates for otherwise treatable HNC, exacerbating both health and economic inequities [2,25]. Vulnerable populations—particularly those with low socioeconomic status or limited insurance coverage—are more likely to experience delays in diagnosis and treatment, leading to advanced-stage presentation, poorer outcomes, and higher cumulative costs [10]. Geographical concentration of specialist services exacerbates these inequalities even further. Long travel distances to referral centers in rural or underserved areas present further hurdles for many patients, limiting timely work-up, surgery, adjuvant treatment, and survivorship care.

#### 3.1.2. Resource Allocation and Cost Effectiveness of Interventions

The rapid evolution of HNC surgery is closely linked to the adoption of advanced technologies, such as robotic systems and microvascular free flaps [26]. However, these innovations demand rigorous evaluation of their cost-effectiveness, particularly in an era of constrained healthcare resources. Rising healthcare expenditures continue to place pressure on payers and institutions to ensure that the benefits of cutting-edge interventions justify their substantial costs. Robotic-assisted surgery—particularly transoral robotic surgery (TORS)—has attracted attention for its potential to reduce morbidity, shorten hospital stays, and improve functional outcomes in oropharyngeal SCC [27]. Randomized controlled trials have reported reductions in tracheostomy rates, feeding tube placements, and inpatient length of stay. However, the acquisition and maintenance costs of robotic platforms—ranging from US $1 million to US $2.5 million upfront, plus annual servicing and training—often exceed those of traditional open or minimally invasive approaches [28,29]. Cost-effectiveness analyses have yielded mixed conclusions: one US study reported an incremental cost-effectiveness ratio (ICER) of US $150,000 per quality-adjusted life year (QALY), exceeding typical willingness-to-pay thresholds [30]. In contrast, European analyses suggest that when TORS enables earlier discharge and fewer complications, the ICER may fall below US $50,000/QALY—underscoring the influence of institutional volume and postoperative care pathways on overall value [31].

Microvascular free tissue transfer remains the gold standard for extensive HNC reconstructions, yet its resource intensity is substantial. These procedures require prolonged operative time, multiple surgical teams, specialized equipment, and extended postoperative intensive care unit (ICU) management. Cost analyses have shown per-case expenditures ranging from US $25,000 to US $40,000, excluding costs associated with complications and readmissions [32]. When indirect costs—such as lost productivity during prolonged convalescence—are factored in, total societal costs may exceed US $60,000 per case. Nevertheless, the superior functional and aesthetic outcomes achieved with microvascular reconstruction, including safer swallowing, improved speech, and enhanced quality of life, can justify the expense under certain models, particularly in high-volume centers that maximize procedural efficiency [30].

A promising strategy to improve cost-effectiveness is the implementation of enhanced recovery after surgery (ERAS) protocols tailored to head and neck patients. ERAS programs standardize perioperative care—incorporating multimodal analgesia, early mobilization, and structured nutritional support—and have been shown to reduce hospital length of stay by 2–4 days and overall hospital costs by 15–30% per patient [33]. When combined with minimally invasive techniques, ERAS can significantly narrow the cost gap between high-technology interventions and conventional care pathways.

Value-based approaches are increasingly integrated into surgical decision-making. Pay-for-performance schemes and bundled payment models incentivize institutions to improve outcomes while controlling costs. In the Netherlands, a bundled payment model for HNC encompassing surgery, radiotherapy, and rehabilitation resulted in a 10% cost reduction over five years, alongside improvements in functional recovery metrics [33]. Similarly, in Canada, population-based analyses indicate that centralized care pathways at accredited centers deliver superior quality at lower per-patient cost [8]. Conversely, high-cost technologies do not uniformly translate into value. For example, intraoperative fluorescence-guided surgery and augmented-reality navigation, while promising for margin delineation, may provide limited incremental benefit relative to their cost—raising concerns about potential overuse without clear cost-effectiveness evidence [34]. In low-resource environments, even fundamental advances such as microvascular anastomosis training may remain inaccessible due to financial and infrastructure constraints [35,36]. Emerging evidence suggests that context-specific solutions can maximize value. Hybrid cancer centers in LMIC have achieved cost benchmarks comparable to those in HIC settings by strategically acquiring refurbished equipment, promoting task-sharing between specialists and trained general surgeons, and adopting selective insurance coverage models [23]. These examples demonstrate that innovation can be accessible without prohibitive expense—provided it is paired with effective systems planning, outcome measurement, and capacity building. Ultimately, allocating resources in HNC surgery requires balancing clinical excellence with fiscal responsibility.

That balance should also include the societal costs borne by patients and families, such as absences from work, travel-related expenses, informal caregiving and diminished earning capacity during survivorship. This means that a perspective purely based on either the provider-side or payer side underappreciates the actual burden of HNC care. Cost-effectiveness is not a fixed attribute of a technology but a dynamic outcome shaped by institutional surgical volume, care pathways, reimbursement mechanisms, and patient selection criteria. Achieving optimal value requires ongoing health-economic evaluation, the adoption of value-based payment models, and context-specific implementation strategies. As the discussion turns to sustainability, it becomes clear that cost-conscious innovation must be aligned with environmental stewardship—developing surgical models that are both economically and ecologically viable (Table 1).

Collectively, these studies support a multidimensional interpretation of economic burden in HNC surgery, extending beyond hospital expenditure to include productivity losses, survivorship-related financial toxicity, and variation in value according to care pathway design.

### 3.2. Healthcare Sustainability

#### 3.2.1. Environmental and Institutional Sustainability

Environmental sustainability is rapidly shifting from a desirable objective to an ethical imperative in HNC surgery. Surgical services—particularly operating rooms (ORs)—are high-impact areas, consuming disproportionately high levels of energy, generating large volumes of waste, and producing significant greenhouse gas (GHG) emissions [36]. Multiple studies estimate that ORs consume three to six times more energy than other hospital areas and generate up to 70% of hospital solid waste [12]. This high resource consumption stems from heavy reliance on single-use equipment, the energy demands of high-temperature sterilization, and the use of anesthetic gases with substantial global warming potential. Several investigations have quantified the plastic waste generated during common procedures such as thyroidectomy and laser laryngeal surgery. One audit found that laryngeal laser cases performed in the OR produced nearly 3 kg of total waste per case, of which only 18% was recyclable. By contrast, performing the equivalent procedure in an office-based setting generated only 10% of that total waste and achieved a recycling rate of 38% [12,17]. Separate studies confirm that approximately 80% of OR waste from HNC surgery is non-recyclable—equating to around 7 kg per case—highlighting the urgent need for improved waste segregation and reuse practices [16,36]. Reducing single-use plastics represents a particularly impactful opportunity. Surveys by ear nose throat (ENT) United Kingdom (UK) indicate that nearly half of outpatient ENT clinics rely exclusively on disposable instruments, with only 11% using reusable alternatives. Transitioning to reusable instruments has the potential to reduce carbon footprints by 38–56%, even when accounting for the additional water and energy required for sterilization [37]. Achieving this transition requires investment in reprocessing infrastructure, comprehensive staff training, and changes in long-standing clinical habits.

#### 3.2.2. Disparities in Access and Financial Burden

Disparities in HNC surgery are shaped not only by patient-level socioeconomic status but also by geography, workforce distribution, language access, insurance design, and differential access to specialist services. In many high-income country (HIC) settings, the greatest sustainability challenge arises from excessive reliance on single-use devices, batch activity in operating rooms and carbon-intensive supply chains. In contrast, numerous LMIC institutions are already using reusable or resterilizable equipment more than single-use items because of cost and supply considerations (Table 2).

Overall, the available evidence indicates that disparities in HNC surgery are not limited to patient-level socioeconomic factors but are also shaped by referral geography, workforce density, language access, insurance design, and the unequal diffusion of specialist services and digital tools. While this minimizes reliance on disposable products, the net environmental benefits can be lost due to lack of reliable sterilization capacity, limited waste segregation mechanisms, less resilient supply chains and aging infrastructure. In HICs, environmentally responsible surgical practice often requires reducing excessive consumption and single-use devices. In LMICs, sustainable surgical policy may depend more on locally appropriate procurement, safe reprocessing, maintenance capacity, and resilient supply chains. Reusable textiles, such as surgical gowns, also offer substantial sustainability benefits. Transitioning from disposable to reusable gowns can reduce energy and water consumption by more than 60%, with a proportional decrease in GHG emissions [16]. Notably, reusable textiles often achieve cost parity with disposable equivalents after just three cycles of use—even without factoring in the additional savings from reduced waste management. Reducing anesthetic-related emissions represents another high-yield intervention. Volatile agents such as desflurane and nitrous oxide have exceptionally high global warming potentials—approximately 2500 and 300 times that of CO_2_, respectively—and are responsible for more than 50% of OR-related carbon emissions [15]. Hospitals in Scotland, the US, and Europe have successfully eliminated desflurane from clinical practice, replacing it with alternatives such as sevoflurane, which substantially lower carbon emissions and can save institutions hundreds of thousands of dollars annually [17]. The adoption of low-flow anesthesia techniques can amplify these environmental and financial gains.

Institutional sustainability, however, extends beyond the choice of equipment and anesthetic agents; behavioral and cultural change is equally critical. Systematic reviews have identified common barriers, including limited staff awareness, inadequate recycling infrastructure, insufficient leadership support, and competing clinical priorities among surgical and anesthetic teams [17]. Survey data from Canadian ENT reveal widespread recognition of climate change (86%), but less than half (46%) acknowledge ORs as significant contributors, and only about 11% report having received formal sustainability education [3]. Aligning staff values with sustainable practices requires structured engagement, including integrating sustainability metrics into training, designating institutional champions, and embedding environmental goals into standard surgical protocols. Quality improvement initiatives offer practical models for change. For example, one Canadian academic department increased e-prescription use from 10% to 40%, reducing emissions by an estimated 57 kg of CO_2_ equivalents while improving provider satisfaction [32]. Similarly, broader UK studies have demonstrated that improved OR sustainability is associated with higher staff and patient satisfaction, enhanced institutional reputation, and potential cost savings [3]. Specific literature for sustainability in HNC surgery is still sparse, but broader principles are easily transferrable: the introduction of reusable devices where safe and practicable, improved waste segregation and recycling practices, optimizing anesthesia gas usage and cultural change through meaningful staff engagement, with measures adapted to the unique contexts of both HIC and LMIC settings.

#### 3.2.3. Workforce and Surgical Capacity

A robust, well-distributed surgical workforce is the cornerstone of sustainable head and neck cancer (HNC) care. Yet human resources in this field remain critically strained worldwide, particularly in LMICs. The Global Otolaryngology–Head and Neck Surgery (OHNS) Workforce survey estimated that many regions have fewer than 0.2 OHNS specialists per 100,000 population—well below the threshold needed to meet local surgical demand [44]. Training disparities mirror these workforce shortages: a recent international study of 91 OHNS practitioners found that two-thirds of respondents from LMICs reported significantly less access to subspecialty training and educational resources compared with their counterparts in HICs, resulting in reduced confidence in performing complex head and neck procedures [44]. This educational inequity limits surgical capacity, leaving few local surgeons trained in advanced techniques such as microvascular reconstruction or skull base surgery—skills essential for comprehensive HNC management [44].

There are important geographic consequences of workforce shortages, as many patients need to travel a long distance to centers capable of delivering comprehensive HNC treatment. While this centralization of expertise may be essential for quality assurance, it results in diagnostic delay and longer time-to-treatment as well as increased out-of-pocket costs and reduced adherence rates with follow-up, especially among rural populations and those experiencing socioeconomic disadvantage.

Global partnerships have become instrumental in narrowing these gaps. Needs assessments in Ethiopia, Kenya, and Zimbabwe highlight a high demand for targeted training in complex reconstructive and airway procedures, with visiting teams from HICs collaborating to transfer skills and establish locally relevant protocols [2]. Success in these initiatives depends on bidirectional engagement—ensuring that both host and visiting teams align priorities, acknowledge infrastructure limitations, and focus on procedures that are sustainable within the local context [2].

Beyond short-term missions, formal academic collaborations have demonstrated substantial impact. For example, partnerships between the College of Surgeons of East, Central and Southern Africa (COSECSA) and the Royal College of Surgeons in Ireland (RCSI) have supported the development of standardized OHNS curricula, rigorous objective examinations, and e-learning platforms, thereby systematically building regional surgical capacity [45]. These programs exemplify ethically grounded, mutually beneficial models that prioritize local leadership and long-term outcome tracking.

The Lancet Commission on Global Surgery has underscored that 5 billion people lack access to safe, affordable surgical care—two-thirds of this unmet need is concentrated in LMICs [2]. Shortages of qualified surgeons, anesthetists, and obstetricians—estimated at over 1.1 million professionals—threaten efforts to scale essential surgical services worldwide [2]. WHO-led initiatives, such as the Global Initiative for Emergency and Essential Surgical Care (GIEESC) launched in 2005, have focused on capacity building through district hospital training toolkits, workforce development programs, and task-shifting policies [46]. Such policies empower nonphysician clinicians to perform essential surgical procedures in settings where specialist coverage is unavailable.

Ensuring training sustainability requires innovative educational modalities. Tele-mentorship and remote case review have proven invaluable during and after the COVID-19 pandemic, enabling continuous education and clinical support without reliance on in-person instruction [47]. Simulation-based training, incorporating low-cost models and virtual platforms, further extends reach and fosters procedural competence [48].

However, workforce expansion must be matched with structural and institutional support. Retention depends on fair remuneration, clear career development pathways, and robust professional networks. Academic partnerships that promote local faculty development, research mentorship, and leadership training—such as those fostered by the Global Surgical Consortium (GSC) founded by Kelly McQueen—strengthen institutional resilience and improve staff retention [49].

#### 3.2.4. Innovation, Digital Health, and Unequal Adoption

The intersection of innovation—through telemedicine, artificial intelligence (AI), and three-dimensional (3D) printing—with HNC surgery is rapidly reshaping clinical practice, training, and access, particularly in underserved regions. These technologies present promising avenues to enhance surgical planning, reduce disparities, and improve both cost-effectiveness and patient-centered care.

Telemedicine, long a key component of HNC surgery follow-up and survivorship programs, mitigates barriers related to geography and socioeconomic status. A U.S. economic assessment reported per-visit savings of US $141 to $223 through virtual consultations, primarily by reducing travel and time away from work—potentially alleviating financial toxicity for underserved patients [50]. Telehealth survivorship initiatives have demonstrated high patient satisfaction and feasibility, effectively monitoring late treatment effects while expanding access to specialized care [51]. Systematic reviews underscore telemedicine’s capacity to improve symptom management, quality of life, and self-efficacy in HNC populations while also emphasizing the need for more rigorous evaluation frameworks [52].

In lower-resource settings, telehealth and AI may remain constrained by limited connectivity, hardware access, workforce capacity, institutional funding, and regulatory support. In this sense, digital solutions should be considered as contextually enablers and not transferable interventions.

In rural or resource-limited settings, AI-augmented telemedicine platforms can provide diagnostic support and remote monitoring, enabling non-specialist health workers to manage complex cases [50]. AI applications in tumor detection and treatment planning for HNC have shown early promise, yet barriers such as clinician trust, limited diversity in training datasets, and unresolved ethical considerations continue to hinder widespread implementation [53].

Moreover, 3D printing and virtual surgical planning have become integral to complex reconstruction and preoperative strategy. A 2024 workflow study demonstrated that establishing an in-house CAD/CAM unit using free software significantly reduced time and costs compared with outsourced services [54]. Additional evidence supports VSP’s benefits in improving surgical precision and enhancing education, allowing trainees to rehearse anatomy and procedures using patient-specific models [54].

Collectively, these innovations contribute to the sustainability of both training and care delivery. Tele-mentoring and remote case reviews—especially during the COVID-19 pandemic—have maintained specialist guidance in LMICs and rural areas [55]. Augmented and virtual reality-based simulators facilitate repetitive skill development even in resource-constrained environments, fostering surgical proficiency [48].

However, the potential of innovation is tempered by risks of deepening inequities if deployment is uneven [11]. AI tools must be trained on diverse datasets to avoid bias against underrepresented populations [53]. Similarly, although the costs of 3D printing and associated software are decreasing, they remain substantial, and regulatory requirements for medical-grade systems can delay institutional adoption [54].

#### 3.2.5. Policy, Reimbursement, and Health System Resilience

The sustainability of HNC surgery services is strongly shaped by policy frameworks and reimbursement structures within health systems.

Cross-country comparison of reimbursement models remains challenging because payment mechanisms, coding systems, insurance design, and hospital financing structures vary substantially across health systems. Consequently, wider economic evaluations that only depend on reimbursement standards in the broader evaluation of HNC care should be combined with important patient-reported outcomes as out-of-pocket costs, productivity loss, caregiver strain, and long-term financial toxicity.

Insurance models, diagnosis-related group (DRG) mechanisms, and policy reforms directly influence access to care, quality of services, and institutional preparedness for future disruptions such as pandemics.

Although reimbursement schemes are integral to creating clinical incentives, aligning them with cost-effective care delivery remains a highly contingent concept largely shaped by the organization of national and regional health systems. In many high-income countries, DRG-based financing is intended to promote efficiency by providing a fixed payment per case. However, if not properly calibrated, these models may inadvertently discourage the adoption of advanced yet costly technologies such as TORS or microvascular reconstruction [56]. For example, financial analyses from Australia found that TORS was under-reimbursed under existing DRG codes, placing significant cost pressures on surgical units despite evidence of improved patient safety and reduced postoperative morbidity [29].

Bundled payment models have emerged as an innovative alternative. Under these arrangements, providers receive a global payment that covers preoperative planning, the index hospitalization, rehabilitation, and follow-up. A European multicenter pilot demonstrated that bundled payments for HNC treatment reduced costs by 10–15% and improved adherence to clinical pathways, without compromising patient satisfaction or functional outcomes [33]. These findings highlight the potential for well-aligned financial incentives to promote high-value care delivery.

Insurance coverage and financial risk protection are equally critical, particularly in LMICs. In Mexico, the Seguro Popular program effectively eliminated out-of-pocket expenses for cancer surgeries, including HNC procedures. Following its introduction, surgical uptake increased by 15%, accompanied by reductions in delayed presentations and emergency interventions [33]. This example illustrates how policy reforms focused on risk pooling and cost-shifting can lead to measurable improvements in access and outcomes.

The COVID-19 pandemic underscored the importance of health system resilience. During the crisis, elective HNC surgeries were often paused for extended periods, revealing systemic vulnerabilities [57]. However, centers with operational reserves—such as established telemedicine systems, cross-trained personnel, and flexible scheduling—were able to resume critical services more rapidly while protecting healthcare workers [58].

Crucially, these are easier to implement in systems with plentiful resources. Flexible staffing and telehealth-supported continuity models may be difficult to sustain in lower-resourced settings with fewer staffing reserves, limited digital infrastructure, and looser financing mechanisms, further emphasizing the need for context-specific resilience planning.

Regulatory support also plays a central role in facilitating innovation. Accelerated approval pathways for robotic platforms or intraoperative navigation systems have lowered barriers to adoption in Europe and North America, provided they are accompanied by robust post-market surveillance [56]. Similarly, the reimbursement codes for telehealth visits introduced during the pandemic have been extended in many jurisdictions, sustaining a 20–30% increase in virtual surgical consultations [51].

Cross-sector collaboration can amplify the impact of policy reform. Engagement among government agencies, professional societies, and patient advocacy groups ensures that reimbursement structures reflect clinical realities. In Canada, a national forum bringing together provincial health ministries, HNC oncologists, and patient representatives successfully incorporated functional outcomes into funding formulas, thereby enhancing access to rehabilitation services [8].

## 4. Conclusions

Sustainable HNC surgery demands an integrated approach that balances clinical excellence with environmental stewardship, equitable access, and system resilience. Evidence shows that targeted measures—such as replacing disposable with reusable materials, optimizing anesthetic practices, and embedding sustainability metrics into training—can substantially reduce resource consumption and greenhouse gas emissions without compromising patient outcomes. Building and retaining a skilled, well-distributed workforce is equally critical, particularly in low- and middle-income countries, where disparities in training and infrastructure remain major barriers to comprehensive care.

Technological innovations such as telemedicine, AI, and 3D printing present an important opportunity to enhance surgical planning, training, and equitable access; however their delivery is currently most feasible in settings with high resource availability and may continue to be inaccessible in many LMICs unless coupled with the necessary infrastructure and human resources investment alongside policy design that prioritizes equity.

Reducing travel-related barriers to specialist care is part of this challenge, as excessive distance to referral centers may translate into delayed treatment, higher out-of-pocket costs, and lower continuity of care.

Policy frameworks and reimbursement structures must align incentives with high-value, patient-centered care, supporting both innovation and long-term sustainability.

Several limitations should be acknowledged. First, as a structured narrative review, this article is susceptible to selection bias. Although a broad search strategy and predefined inclusion criteria were used, the review was not designed to identify or appraise all relevant studies exhaustively. Second, the included literature was heterogeneous in study design, populations, outcomes and geographical settings, which limits comparability between studies and precludes a formal quantitative synthesis. Furthermore, this review did not systematically compare surgery with non-surgical modalities (radiotherapy or systemic therapy) and cannot assess secular trends in surgical volume and cost over time—all of which warrant focused comparative studies and registry-based research as a priority for future investigation.

Third, numerous themes in this review—especially sustainability practices, reimbursement models and digital innovation—are closely linked to the organization of local health systems, which may limit potential generalizability of single research results. Accordingly, the findings should be interpreted as a conceptual and integrative synthesis of representative evidence, not as a pooled estimate of effect or a formal assessment of certainty.

Embedding the principles of sustainable development into every link across the entire continuum of surgical care for head and neck cancers—including referral, procurement, surgical care, and other related stages—can strengthen healthcare service capacity while securing equitable, high-quality medical care for both current and future patients. This requires coordinated action across clinical, technological, and policy domains, underpinned by global collaboration and a commitment to health equity. Future research should include a dedicated systematic review, ideally following PRISMA methodology, to comprehensively map socioeconomic determinants of HNC surgery, assess the methodological quality of available evidence, and identify gaps requiring prospective, registry-based, or administrative-data studies. Such work should integrate episode-of-care costing across surgery, radiotherapy, and systemic therapy with patient-reported financial toxicity, productivity losses, QALY-based outcomes, and procedure-level environmental metrics.

## Figures and Tables

**Table 1:** High and medium narrative appraisal categories reflect qualitative judgments based on study design, methodological transparency, relevance to HNC surgery or surgical systems, and consistency with the review framework. They are not formal certainty-of-evidence ratings.

Author	Year	Study Design	Economic Domain	Key Findings	Main Implication	Narrative Quality Appraisal
Patterson et al. [1]	2020	Global economic burden analysis	Macroeconomic burden	HNC was associated with major worldwide productivity losses and long-term economic impact, especially in LMICs	Supports inclusion of societal and productivity losses in HNC economic evaluation	High
Smith et al. [3]	2023	Retrospective cohort/financial toxicity study	Direct and patient-level costs	Patients with HNC experienced substantial out-of-pocket costs and financial toxicity during treatment and survivorship	Financial toxicity should be considered a core outcome in HNC care models	High
Baddour et al. [4]	2021	Survivorship cost study	Objective and subjective financial toxicity	Long-term HNC survivorship was associated with persistent financial strain	Follow-up pathways should incorporate financial navigation and survivorship support	High
Silfverschiöld et al. [7]	2024	Cost-of-illness study	National direct and indirect costs	In Sweden, indirect costs accounted for a large proportion of total HNC burden	Economic models should capture productivity loss, not only hospital expenditure	High
Rodin et al. [29]	2017	Cost-effectiveness analysis	Surgery vs. non-surgical comparator	The cost-effectiveness of TORS versus radiation therapy depended on assumptions regarding outcomes and resource use	Comparative economic evaluation is essential before adoption of high-cost technologies	High
Tom et al. [33]	2019	Clinical/economic analysis	Bundled payment implications	Cost variation in HNC was influenced by clinical complexity and care pathway factors	Supports episode-of-care costing and bundled payment design in HNC	High
Hamilton et al. [37]	2014	Cost–utility review	Otolaryngology cost–utility methods	Cost–utility evidence in otolaryngology remained limited and methodologically heterogeneous	Reinforces the need for more standardized QALY-based economic studies in HNC	Medium

**Table 2:** Representative evidence on healthcare disparities, access barriers, and system-level determinants in HNC surgery.

Author	Year	Study Design	Disparity Domain	Key Findings	Main Implication	Narrative Quality Appraisal
Smith et al. [3]	2023	Retrospective cohort study	Socioeconomic status	Lower socioeconomic status was associated with worse stage at presentation and poorer outcomes	Supports targeted screening and support strategies for vulnerable groups	High
Goodnight et al. [38]	2025	Geospatial analysis with multivariate regression	Geographic access	Rural patients traveled substantially farther for specialist HNC care; greater distance was associated with treatment delay	Supports hub-and-spoke pathways and telehealth-enabled perioperative care	High
Chen et al. [39]	2023	Mixed-methods study	Language barriers	Non-English-speaking patients experienced longer diagnostic intervals and communication barriers	Strengthens the case for interpreter services and culturally adapted communication	Medium
Xu et al. [40]	2022	Cost analysis with patient interviews	Rural financial toxicity	Rural patients incurred higher out-of-pocket costs and more follow-up-related financial distress	Financial navigation and care coordination should be integrated into HNC pathways	Medium
Barnes et al. [41]	2021	Interrupted time series analysis	Insurance/Medicaid expansion	Expanded coverage was associated with reduced cancer mortality and improved access to care	Policy-level risk protection may improve HNC access and outcomes	High
Washington et al. [42]	2025	Propensity-matched analysis	Racial disparity in treatment allocation	Minority patients were more likely to undergo more aggressive laryngeal surgery and had worse survival	Highlights inequity in access to organ-preserving treatment pathways	High
Pozin et al. [43]	2024	Geographic workforce/access analysis	Specialist distribution	Rural and lower-resource areas had poorer otolaryngology access	Workforce distribution is a key structural determinant of HNC care inequity	Medium
Petrucci et al. [44]	2023	Global workforce survey	Workforce shortage	Many regions had extremely low OHNS specialist density, especially in LMICs	Capacity building is essential for equitable surgical access	High
Castro et al. [8]	2025	Concept/review on regionalization	Centralization and referral systems	Regionalized HNC care may improve outcomes but may also increase travel burden if not supported appropriately	Centralization should be paired with referral support and access mitigation strategies	Medium
Igwe et al. [11]	2025	Access/telemedicine barriers study	Digital inequity	Digital health opportunities are limited by infrastructure and social barriers in underserved populations	Telehealth should be deployed with equity safeguards	Medium

## Data Availability

No new data were created or analyzed in this study. Data sharing is not applicable to this article.

## Abbreviations

The following abbreviations are used in this manuscript:

AI	Artificial Intelligence
COSECSA	College of Surgeons of East, Central and Southern Africa
DRG	Diagnosis-related group
ENT	Ear Nose Throat
ERAS	Enhanced recovery after surgery
GHG	Greenhouse gas
GIEESC	Global Initiative for Emergency and Essential Surgical Care
GSC	Global Surgical Consortium
HIC	High-income countries
HNC	Head and Neck Cancer
ICER	Incremental cost effectiveness ratio
ICU	Intensive Care Unit
LMIC	Low- and Middle-Income Countries
OHNS	Otolaryngology–Head and Neck Surgery Workforce
OR	Operative room
QALY	Quality-adjusted life year
RCSI	Royal College of Surgeons in Ireland
SCC	Squamous Cell Carcinoma
TORS	Trans Oral Robotic Surgery
UK	United Kingdom
US	United States
WHO	World Health Organization
3D	Three-dimensional
